# Modelling the within-herd transmission of *Mycoplasma hyopneumoniae* in closed pig herds

**DOI:** 10.1186/s40813-016-0026-1

**Published:** 2016-04-01

**Authors:** Heiko Nathues, Guillaume Fournie, Barbara Wieland, Dirk U. Pfeiffer, Katharina D. C. Stärk

**Affiliations:** 1 Veterinary Epidemiology, Economics and Public Health Group, Royal Veterinary College London, Hawkshead Lane, Hatfield, Hertfordshire AL97TA UK; 2 Clinic for Swine, Vetsuisse Faculty, University of Berne, Bremgartenstrasse 109a, 3012 Bern, Switzerland; 3 International Livestock Research Institute, Addis Ababa, Ethiopia

**Keywords:** Enzootic pneumonia, Infectious disease, Epidemiology, Prevention

## Abstract

**Background:**

A discrete time, stochastic, compartmental model simulating the spread of *Mycoplasma hyopneumoniae* within a batch of industrially raised pigs was developed to understand infection dynamics and to assess the impact of a range of husbandry practices. A ‘disease severity’ index was calculated based on the ratio between the cumulative numbers of acutely and chronically diseased and infectious pigs per day in each age category, divided by the length of time that pigs spent in this age category. This is equal to the number of pigs per day, either acutely or chronically infectious and diseased, divided by the number of all pigs per all days in the model. The impact of risk and protective factors at batch level was examined by adjusting ‘acclimatisation of gilts’, ‘length of suckling period’, ‘vaccination of suckling pigs against *M. hyopneumoniae*’, ‘contact between fattening pigs of different age during restocking of compartments’ and ‘co-infections in fattening pigs’.

**Results:**

The highest ‘disease severity’ was predicted, when gilts do not have contact with live animals during their acclimatisation, suckling period is 28 days, no vaccine is applied, fatteners have contact with pigs of other ages and are suffering from co-infections. Pigs in this scenario become diseased/infectious for 26.1 % of their lifetime. Logistic regression showed that vaccination of suckling pigs was influential for ‘disease severity’ in growers and finishers, but not in suckling and nursery pigs. Lack of contact between gilts and other live pigs during the acclimatisation significantly influenced the ‘disease severity’ in suckling pigs but had less impact in growing and finishing pigs. The length of the suckling period equally affected the severity of the disease in all age groups with the strongest association in nursery pigs. The contact between fatteners of different groups influenced the course of infection among finishers, but not among other pigs. Finally, presence of co-infections was relevant in growers and finishers, but not in younger pigs.

**Conclusion:**

The developed model allows comparison of different prevention programmes and strategies for controlling transmission of *M. hyopneumoniae*.

**Electronic supplementary material:**

The online version of this article (doi:10.1186/s40813-016-0026-1) contains supplementary material, which is available to authorized users.

## Background

*Mycoplasma hyopneumoniae* is the primary pathogen of porcine enzootic pneumonia (EP). The occurrence, the course and the severity of EP in pigs harbouring *M. hyopneumoniae* in their respiratory tract is influenced by a number of factors such as virulence of the particular strain [1] as well as the additional co-infections with other respiratory pathogens and miscellaneous risk factors [2]. *M. hyopneumoniae* is introduced into a herd either by direct transmission following the purchase of infected pigs, or by airborne transmission [3]. Subsequently, the within-herd transmission is maintained vertically by nose-to-nose contact between sows and their offspring [4] or by horizontal route between pen mates or pigs in the same compartment [5]. If an all-in/all-out flow of pigs is not consequently implemented between production stages, transmissions of *M. hyopneumoniae* from infected older to naïve younger pigs is likely [2]. In general, pigs of every age can become infected, although in endemically infected farms mature pigs usually serve only as a reservoir for the pathogen, whereas growing pigs more often develop clinical signs of EP. For the infection of pigs with *M. hyopneumoniae* and the corresponding disease several risk factors, e.g. poor management practices, co-infections with other bacteria, viruses and/or parasites, seasonal effects, have been described [4, 6–9]. Some studies examined the role of suckling and nursery pigs and their individual risks for positivity to *M. hyopneumoniae.* Authors found that the presence of the porcine reproductive and respiratory syndrome virus (PRRSv)-EU genotype, *Pasteurella multocida*, *Haemophilus parasuis*, *Mycoplasma hyorhinis* or *Streptococcus suis* in the lung tissue of nursery pigs was significantly correlated with a higher probability of also finding *M hyopneumoniae,* whereas sow parity was not statistically related with piglet colonization in the offspring [10, 11]. Other studies focused on prevalence within different age groups [12] or follow-up of infected piglets [13], thus providing crucial knowledge for a better understanding of spread of *M. hyopneumoniae* in pig herds. Improved housing and management conditions are essential part of strategies for controlling EP [2]. Moreover, vaccination can reduce the impact of disease in endemically infected herds [14], but does not eliminate the pathogen from an infected herd [15].

In recent years, mathematical models of infectious diseases in animal populations have been widely used to gain insights about disease dynamics and the impact of control interventions. Mathematical models have, for instance, been used to enhance our knowledge about the dynamics of Methicillin-resistant *Staphylococcus aureus* [16] PRRSv [17], *Salmonella Typhimurium* [18] and transmissible gastro-enteritis in pig herds [19]. Through the identification of factors and interventions, they can be used by animal-health stakeholders – including policy-makers, veterinarians and farmers – as a decision-support tool. Models require data in order to be parameterised. In the case of *M. hyopneumoniae* a significant amount of information has been published over the last years, including the basic reproduction number (R_0_) in different age groups, incubation period, etc. However, to the authors´ knowledge, no mathematical model has yet described the course of EP in a closed pig herd.

Here we use a compartmental model simulating the spread of *M. hyopneumoniae* within a batch of indoor and intensively raised pigs to assess the impact of a range of husbandry practices, industry settings and control interventions on the occurrence and spread of the pathogen. Therefore, a ‘disease severity index” was calculated based on days when pigs were acutely or chronically diseased and infectious. The aims of developing this model were gaining insights about disease dynamics and comparing different prevention programmes and strategies for controlling EP. Finally, this model shall help veterinarians and farmers as support tool in their decision making process.

## Methods

### Model design

In the present study, a discrete time, stochastic, compartmental model was developed, where one time-step equalled to one day. The unit of the model was the individual pig and a specific closed production batch of pigs was modelled from their birth to slaughter considering demographics of a pig population, including deaths. All piglets were born on the same day and each pig successively passed four age categories: suckling, nursery, growing and finishing. The time spent by pigs in each age category was fixed to 21 or 28 days of suckling period and 49 or 42 days of nursery period, respectively, 28 days of growing and 82 days of finishing. All pigs in a given batch were moved from one age category to the next together. For simplification, random mixing of all animals within each batch was assumed.

**Fig. 1:**
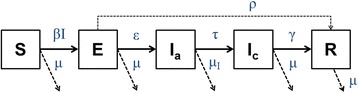
Conceptual design of the compartmental model for transmission of *M. hyopneumoniae* within pig herds. (S: susceptible, E: exposed, Ia: acutely diseased and infectious, Ic: chronically diseased and infectious, R: recovered; transition parameters are explained in Table 1)

To model infection in the herd five successive states, i.e. compartments, were defined: susceptible (S), exposed or pre-infectious (E), acutely diseased and infectious (I_a_), chronically diseased and infectious (I_c_), and recovered (R) (Fig. 1). Given that there is no intra-uterine transmission [20], all suckling pigs were considered susceptible (S) after birth. In endemically infected pig herds, in which sows are frequently seropositive to *M. hyopneumoniae*, new-born suckling pigs will obtain a varying amount of maternally derived antibodies, but these do not protect against infection thus leaving the piglets fully susceptible [4]. Once infected, pigs are defined as being exposed, or pre-infectious (E) which means they are asymptomatic and do not shed the pathogen. At onset of clinical signs (coughing, etc.), pigs were considered as ‘*acutely diseased and infectious’* (I_a_), and thereby beginning to shed the pathogen and therefore allow spread to susceptible pigs. Following this period, pigs became ‘*chronically diseased and infectious’* (I_c_); a state in which pigs do no longer show clinical symptoms but still shed the pathogen. Finally, pigs were considered to recover from the infection. These animals will have developed specific immunity [21] and no longer contributed to the transmission of *M. hyopneumoniae* within the herd. Some pre-infectious pigs were considered directly recovering from infection without experiencing symptoms or shedding the pathogen.

A pig could die at any time step during the batch cycle with its probability of survival depending on the age and the health state. The number of pigs in age category *i* in a given health state, surviving between time *t* and *t* + 1, was simulated by a binomial process with the number of pigs in a given health state at time *t* as the number of trials, and the probability of survival ($\mathsf{1}-{\mathit{\mathsf{\mu}}}_{\mathit{\mathsf{I}},\mathit{\mathsf{i}}}$ for *I*_*a*_ and $\mathsf{1}-{\mathit{\mathsf{\mu}}}_{\mathit{\mathsf{i}}}$ for all other health states) as the probability of a success. Likewise, the transitions between infection states were simulated using binomial processes. The probability *p*_*i*,*t*_ of a susceptible pig at time *t* in age category *i* becoming infected at time *t + 1* was dependent on *I*_*a*,*i*,*t*_, *I*_*c*,*i*,*t*_, *N*_*i*,*t*_ and *β*_*i*_ (Eq. 1). A special situation was given for suckling pigs, which were infected by gilts and sows only. The probability of a piglet being infected by a sow increased with the duration of the suckling period. It was modelled using an exponential function (Eq. 2). The function parameters were selected in order to reproduce observed prevalence of *M. hyopneumoniae* infections in suckling pigs [22].

1 $$ {p}_{i,t}=1- exp\left\{-{\beta}_i\left({I}_{a,i,t}+\alpha {I}_{c,i,t}\right)/{N}_{i,t}\right\} $$

*I*_*a*,*i*,*t*_, *I*_*c*,*i*,*t*_ and *N*_*i*,*t*_ were the number of acutely diseased and infectious pigs, chronically diseased and infectious pigs and the total number of pigs in age category *i* at time *t*, respectively. *β*_*i*_ was the rate of transmission for acutely diseased and infectious pigs in age category *i* and *α* was the relative infectivity of chronically compared with acutely diseased and infectious pigs, which was 0.5 by default.

2 $$ {p}_{suckling,t}=0.000251*\ {e}^{0.1t} $$

*t* was the number of time-steps. As all piglets were born at *t* = 0, it was also interpreted as the age, in days, of the suckling pigs.

The length of time that pigs spent in each infection compartment (E, I_a_ and I_c_) followed a normal distribution. The probability of a pig leaving an infection compartment increased with the time already spent in that compartment and was given by the cumulative distribution function (Table 1). As mentioned above, pigs leaving the E compartment could either become acutely diseased and infectious or recover from the infection. The number of pigs leaving the compartment E and directly recovering was simulated by a binomial process with the number of pigs leaving the compartment E as the number of trials and the probability *ρ* of not getting diseased and infectious following exposure as the probability of a success.

**Table 1:** Values of different, partly age-dependent transition parameters for a discrete time, stochastic compartment model estimating the within-herd transmission of *Mycoplasma hyopneumoniae* in closed pig herds

Parameter	Age group	Parameter level	Source
β	Suckling pigs	0.0005	[13]
	Nursery pigs	0.0148	
	Growing pigs	0.1497	
	Finishing pigs	0.1497	
ε	Nursery pigs	NCDF*(18, 7)	[41]
	Nursery pigs		
	Growing pigs	NCDF(13, 1.5)	[42]
	Finishing pigs		
τ	All pigs	NCDF(14, 3.5)	[42]
γ	All pigs	NCDF(28, 7)	[21]
ρ	All pigs	0.200	Expert opinion
μ	Suckling pigs	(0.02*e^(−0.233*x)^) + 0.002	[23]
	Nursery pigs	((2.0/100)/t_Nursery period_)	
	Growing pigs	((1.0/100)/t_Growing period_)	
	Finishing pigs	((1.6/100)/t_Finishing period_)	
μ_I_	Suckling pigs	μ _[Suckling pigs]_	
	Nursery pigs	μ*2	
	Growing pigs	μ*2	
	Finishing pigs	μ*2	

*NCDF: normal cumulative distribution function with (μ, σ)

Table 1 describes transition parameters and other input parameters of the model.

### Input parameters & outcome variable

#### Population dynamics parameters

Numbers corresponding to a one-site production system with approximately 500 producing sows and their offspring originating from a weekly batch farrowing were calculated in order to fit the model with representative numbers for an average sized herd. Based on these numbers, a standardized herd consisted of 21 farrowing groups of approximately 24 sows each (Eq. 3).

3 $$ \mathrm{Number}\ \mathrm{of}\ \mathrm{sows}\ \mathrm{per}\ \mathrm{group}\ \left(\mathrm{N}\right)=\frac{\mathrm{Number}\ \mathrm{of}\ \mathrm{sows}\ \mathrm{in}\ \mathrm{the}\ \mathrm{herd}\ \left(\mathrm{n}\right)}{\left[\frac{\mathrm{Length}\ \mathrm{of}\ \mathrm{gestation}\ \left(\mathrm{weeks}\right)+\mathrm{suckling}\ \mathrm{per}\mathrm{iod}\ \left(\mathrm{weeks}\right)+\mathrm{dry}\ \mathrm{per}\mathrm{iod}\ \left(\mathrm{weeks}\right)}{\mathrm{Farrowing}\ \mathrm{rhythm}\ \left(\mathrm{weeks}\right)}\right]} $$

Production parameters, e.g. number of life born piglets per litter or suckling pig mortality, were based on the annual report on pig production in Germany [23]. The number of piglets born live per batch was set at 293 (24 sows * 12.2 piglets born live per litter). The average proportion of pigs dying during the suckling period was 14.8 % [23], with approximately 50 % of the deaths occurring in the first four days of life [24]. Therefore, the probability of a suckling pig dying varied with the number of time steps *x* spent in the suckling section, and was expressed as: *μ*(*x*) = 0.002 + 0.02*e* ^− 0.233*x*^. Of pigs entering the nursery, growing and finishing sections, 2.0 %, 1.0 % and 2.1 % died in that section, respectively. The daily rate of death μ for each of these periods was calculated by dividing the percentage of mortality during the particular period by the number of days spent in this period. The lengths of the different production periods in the standard setting (baseline) were empirically set to 28 days for suckling (according to 91/630/EEC), 42 days for nursing, 28 days for growing and 82 days for finishing. In other scenarios, which were also analysed using the model, the suckling period was shortened to 21 days and the nursery period was then extended to 49 days.

#### Transition parameters

The parameters influencing the probability of moving to the next compartment were extracted from the literature, where available. For missing data, parameters were calculated from published data describing the course of *M. hyopneumoniae* infections in different age groups of pigs as described below, or were estimated based on expert opinion. For the expert opinion, 15 specialists for *M. hyopneumoniae* (5 clinicians, 5 microbiologists and 5 epidemiologists)*,* known to the first author, were invited by email to complete an online survey. The survey closed 14 days after the invitation emails had been send. It included three semi-closed questions and one open-ended question:

- Please, imagine a case of *M. hyopneumoniae* infection with a strain of low (question 1), moderate (question 2) or high virulence (question 3):
*‘What do you think is the likelihood for an individual pig to recover from the infection without becoming infectious (= shedding of the pathogen)? Please provide your answer in % (0–100).’*
- What do you think is the average impact of common co-infections (e.g. PRRSV, SIV & PCV2) on the transmission rate of *M. hyopneumoniae*?
*‘When co-infections are present, the transmission rate will increase by 0 %, 10 %, 20 %, 50 %, 100 % or 200 % (This question was to be answered in a table format for ‘suckling pigs’ , ‘nursery pigs’ , ‘growing pigs’ and ‘finishing pigs’)’*

Parameters were adjusted to the particular age group of pigs.

The *β* for each age group (Table 1) was calculated from observed increases of prevalence in a longitudinal study [13]. It was assumed that *M. hyopneumoniae* prevalence (determined by PCR in bronchoalveolar lavage fluid and nasal swabs) reached 6.3 % at the end of the nursing period, 45.9 % at the end of the growing period, and 83.5 % at the end of the fattening period. Following these assumptions, the *β* for each group was calculated considering the overall transmission of the infection in the particular period and the length (D) of the particular infectious period (Eq. 4). The probability *ρ* of not becoming infectious following exposure was determined using expert opinion. Values of *β* were chosen to reproduce these empirical prevalences. In practice, for each successive age group, a wide range of values of *β* were tested. For each value of *β*, the spread of the pathogen within a given age group was simulated 1000 times. The average simulated prevalence of infection at the end of this production period was computed. The value of *β* associated with the average simulated prevalence that was the closest to the observed prevalence was then selected.

4 $$ \beta =\frac{{\mathrm{R}}_0}{\mathrm{D}} $$

In every iteration, a batch started with 293 suckling pigs. Subsequently, pigs could become exposed with *α* probability described in equation 1. However, *β* was dependent on the age group and increased, whenever pigs moved into the next age group. Pigs exposed to *M. hyopneumoniae* could become ‘acutely infectious and diseased’ with a probability of *ε*. The *ε* depended on the time that pigs already spent in that compartment, and was defined a normal cumulative distribution function, as described in the Table 1. Thus, the latent period was normally distributed. Instead of becoming ‘acutely infectious and diseased’, the pigs could also die with a probability *μ* or they could recover with a probability of *ρ*. The lengths of time that pigs remained in the infectious compartments were normally distributed. The probability *τ* and *γ* of an ‘acutely infectious and diseased’ pig becoming ‘chronically infectious and diseased’ , and the probability of a ‘chronically infectious and diseased’ pig recovering from infection followed a normal cumulative distribution function (Table 1). Independent of the infection compartment pigs moved in the age categories from the suckling period (21/28 days) to the nursery period (42/49 days), the growing period (28 days) and finally the finishing period (82 days).

Depending on the virulence of a particular *M. hyopneumoniae* strain, exposed pigs would move directly to the compartment of recovered pigs [25]. The model was parameterised to reflect transmission of a *M. hyopneumoniae* with a substantial level of virulence. Therefore, the value for ρ was assumed low, which was also in accordance with expert opinion.

#### Outcome variable

For each simulation and age category, a disease severity index (S_Disease,i_; Eq. 5) was calculated. It was defined as the ratio between the cumulative numbers of acutely and chronically diseased and infectious pig-days in an age category *i*, divided by the length of time that pigs spent in this age category *i*. This is equal to the number of pigs per day, either acutely or chronically infectious and diseased, divided by the number of all pigs per all days in the model (theoretical maximum is close to 100 %).

5 $$ {S}_{Disease,i}={\displaystyle \sum_{t=0}^{T_i}}\left({I}_{a,i,t}+{I}_{c,i,t}\right)/{T}_i $$

*T*_*i*_, was the length of time in days that pigs spent in an age category *i*.

### Evaluated scenarios

The impact of different risk and protective factors on the spread of the *M. hyopneumoniae* at batch level was examined by adjusting the affected model parameters in the baseline model outlined above.

#### Acclimatisation of gilts (Acc)

A recent study [26] showed that one-site pig production systems are 10 times more likely to suffer from infection with *M. hyopneumoniae* followed by EP, if gilts in the particular herd do not have contact with living pigs of any age during their acclimatisation period. This risk factor was considered as a multiplier of the probability of suckling pigs becoming infected by their dam in case that no appropriate acclimatisation for gilts is implemented in the model herd. The *β* for suckling pigs (SP) was multiplied by 10 in order to account for this increase in the probability of the transmission of *M. hyopneumoniae* from sows and gilts to suckling pigs.

#### Length of suckling period (Suc)

The likelihood of transmission of *M. hyopneumoniae* from sows to their offspring increased exponentially with the length of the suckling period, which is equal to the time under exposure [22, 26, 27]. Two scenarios assuming a length of the suckling period equal to 21 and 28 days, respectively, were tested. The probability of a susceptible pig being infected by a sow on day *d* of its suckling period was equal to 0.000251 * *e* ^0.1*d*^. This likelihood has been calculated considering the negativity of sucking pigs for *M. hyopneumoniae* at birth, a prevalence of 3.5 % at 28 days of age [11] and an average prevalence for the whole suckling period of less the 2 % [10]. The corresponding data were tabled and parameters that best fit these data were selected.

#### Vaccination of suckling pigs against M. hyopneumoniae (Vac)

To assess impact of vaccination no special compartment for vaccinated pigs was included, since vaccination does not protect against infection [28], but with vaccinated piglets, the rate of spread of *M. hyopneumoniae* might be lower [29]. This change in the infection dynamics was considered in the model by lowering *β* by approximately 20 % for the age groups “suckling pigs” and “nursery pigs” in the model [30, 31], when it was assumed that suckling pigs had been vaccinated (16 scenarios out of 18).

#### Contact between fattening pigs of different age during restocking of compartments (Con)

The contact between pigs of different age during restocking of fattening compartments has been shown to promote the spread of the infection in this age group (OR: 13.8; [26]). In order to account for this effect, the models allowed contacts, over one day (i.e. one time-step), between outgoing finishing pigs (i.e. ending their production cycle) and growing and other finishing pigs. It created opportunities for transmission of infection from these outgoing finishing batches to batches in their growing or finishing period. This event could happen at any time during the fattening period. On the day that such contacts occurred, the probability of a pig in its growing or fattening period becoming infected was equal to:

$$ {p}_{i,t}=1- exp\left\{-{\beta}_i\left({I}_{a,i,t}+\alpha {I}_{c,i,t}+{I}_{a,O,t}+\alpha {I}_{c,O,t}\right)/\left({N}_{i,t}+{N}_{O,t}\right)\right\} $$

Where the subscript O denoted the batch of outgoing fattening pigs. When accounting for such contacts between pigs of different age groups, successive production batches were modelled, and transmission of infection between a given batch of fattening pigs ending its production cycle, and subsequent batches of pigs which were in their fattening or growing period was simulated. A total of 100 successive batches was simulated in order to reach a stable prevalence at the end of batch production cycles, i.e. an equilibrium.

#### Co-infections in growing and finishing pigs (Inf)

Knowledge about the impact of co-infections on *M. hyopneumoniae* with regard to transmission of the infection, duration and severity of the disease is rare. Again, therefore expert opinion was used to assess the impact of co-infections as a multiplying factor for *β* in growing and finishing pigs.

Overall nine out of 15 experts answered to the questions regarding the impact of co-infections on the transmission rate of *M. hyopneumoniae*. Their estimate for suckling pigs was ranging from 0 % to 200 % increase and for nursery, growing and finishing pigs it was ranging from 10 % to 200 % increase of the transmission rate. The corresponding median values per age group were 10 %, 20 %, 50 % and 50 %, respectively. Thus, the individual values for *β* per age group were multiplied by 1.1, 1.2 or 1.5 in such scenarios, where the presence of co-infections was hypothesised.

### Validation

The model was validated by comparing the estimated proportions of infected pigs at different stages of the batch production cycle with published figures on prevalence of *M. hyopneumoniae* in endemically infected herds at the same point in time [4, 32, 33]. Average prevalence of infection after 1000 iterations were analysed and compared to the prevalence known for that age category. Moreover, the ranges obtained after 1000 iterations, including minimum and maximum, were analysed for plausibility. Thus, length of the time-period for validation was one batch cycle, usually lasting about 180 days. A simulation with 2 % exposed suckling pigs at the end of the suckling period was used to determine the baseline of infection in the herd of the developed model. Reasons for using exactly this level of exposed piglets were observations made in different studies investigating the prevalence of *M. hyopneumoniae* in this age group [10, 11].

### Sensitivity analysis

A univariate sensitivity analysis was conducted on a subset of transition parameters only to allow for realistic computing time. The selected parameters β and ρ were assumed to be of particular biological importance and, thus, they were multiplied with values between 0.2 and 2.0 in order to simulate only 20 % and up to 200 % of their impact on the model. The outcome variables for multiple comparisons were the ‘severity of disease’ and the proportion of 1,000 iterations not leading to any spread of infection in the herd.

The model was coded and run in R (Version x64-2.15.1; R Core Team (2014)) using TinnR as an graphic user interface [25]. The R programming code of the model is available from the corresponding author upon request. The relative importance of each risk or protective factor for the ‘disease severity index’ in the different age groups was determined by developing a regression model with STATA/IC 12.0 for Windows [64-bit x86–64] (StataCorp LP, Texas, USA). In this step, the disease severity indices of all iterations except of the first 100 were analysed for their association with the presence of risk and protective factors, i.e. the impact of the risk and protective factors on the numeric values in each age category.

## Results

### Validation

**Fig. 2:**
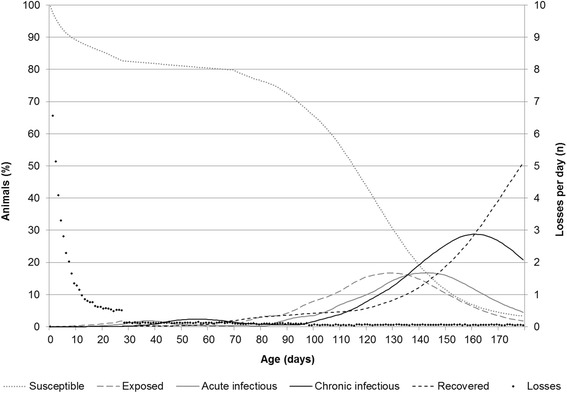
Line chart describing the most likely temporal pattern of *M. hyopneumoniae* infection in a batch of 293 pigs, when three protective factors are all absent (Vac[−], Acc[−], Suc[−]) and two risk factors are both present (Con[+], Inf[+]). Lines represent the average of 1,000 iterations of the stochastic compartment model

**Fig. 3:**
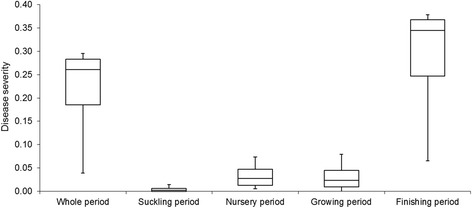
Box plots describing the severity of *M. hyopneumoniae* infection (number of pigs per day either acutely or chronically infectious and diseased divided by the number of all pigs per all days in the model) in a batch of 293 pigs, when three protective factors are all absent (Vac[−], Acc[−], Suc[−]) and two risk factors are both present (Con[+], Inf[+]). Values represent results of 1,000 iterations of the stochastic compartment model. The disease severity is a ratio between the number of pigs per day being infectious divided and the number all pigs per day in the model

The outcomes of various scenarios were plotted as line charts and compared with recently published data on the prevalence of *M. hyopneumoniae* infection in pigs at different age. When all protective factors were present ([P] = Positive; Vac[P], Acc[P], Suc[P]) and risk factors were absent ([N] = Negative; Con[N], Inf[N]), the percentage of finishing pigs susceptible to *M. hyopneumoniae* at the end of the fattening period was >85 % on average and the percentage of pigs, which had been infected during their growth period was <10 % (mean; evidenced by details in Table 2 and ‘Additional file 1’). Mortality that can be observed in this scenario is attributed to ‘baseline mortality’, which is approx. 15 to 20 % from birth to slaughter [23]. The observation of less than 10 % potentially seropositive pigs (due to exposure to *M. hyopneumoniae* followed by latency until seroconversion = I_a_ + I_c_ + R) and the absence of biologically significant within-herd transmission in most simulations are consistent with findings of a recent study [26]. In the latter, no spread of the pathogen and no disease could be confirmed in well-managed pig herds. In such scenarios, all animals should be seronegative at the end of the fattening period, because of waning of maternally derived antibodies, waning of antibodies after vaccination and the absence of exposure to the pathogen. In contrast, nearly all susceptible pigs became exposed and subsequently infectious (Fig. 2), when all protective factors were absent (Vac[N], Acc[N], Suc[N]) and all risk factors were present (Con[P], Inf[P]). The ‘disease severity’ in this ‘high risk’ scenario (Fig. 3) was well in accordance with findings in the field, where herds with similar risk and protective factors show comparable results in terms of pathogen transmission [26]. In contrast, the ‘disease severity’ was negligible in the ‘low risk’ scenario. Details can be studied in a graph provided as ‘Additional file 2’.

**Table 2:**
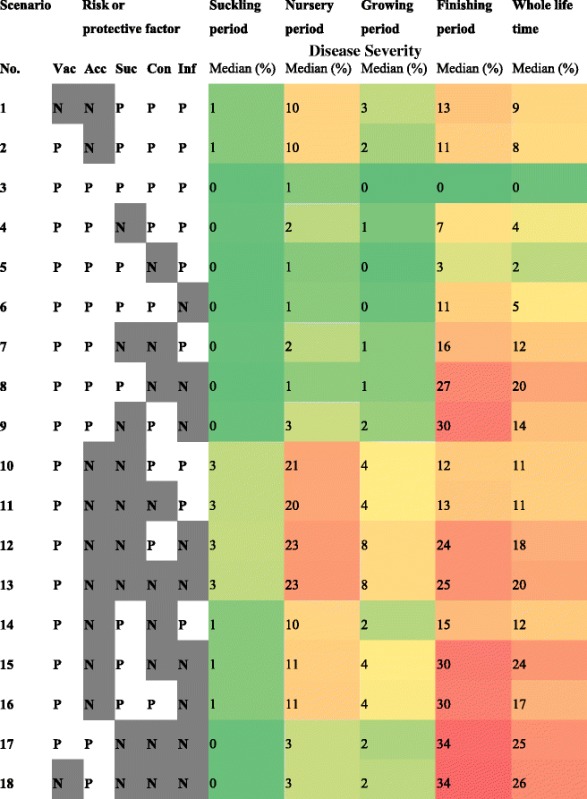
Numerical results of disease severity (number of pigs per day either acutely or chronically infectious and diseased divided by the number of all pigs per all days in the model, given in per cent) and heat map for 18 different scenarios of a compartmental mathematical model of within-herd transmission of *M. hyopneumoniae*

Vac (P): vaccination of suckling pigs against *M. hyopneumoniae*. (N): no vaccination

Acc (P): gilts have contact to living animals during their acclimatisation. (N): no contact to living animals

Suc (P): duration of suckling period is 21 days. (N): suckling period is extended to 28 days

Con (P): growers have no contact to finishing pigs during restocking of compartments. (N): contact between different age groups

Inf (P): pigs do not suffer from co-infections. (N) presence of co-infections

*Severity of disease is defined as the average proportion of days that each pig is acute or chronic infectious during a particular period (e.g. only nursery period. only fattening period or whole life time)

### Sensitivity analysis

The estimation of the model outcomes was based on 1000 iterations, which was considered sufficient since the moving-average disease severity index (mean of last 100 values) was stable after this number of iterations. More details regarding the convergence of the outcome due to the necessity of stabilizing the effect of ‘contact between fattening pigs of different age during restocking of compartments’ are displayed in a graph, which is provided as ‘Additional file 3’.

**Fig. 4:**
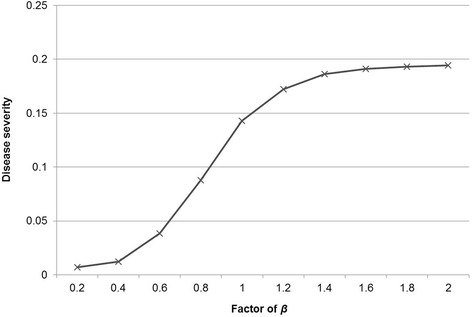
Evaluation of the sensitivity of the outcome variable (disease severity = number of pigs per day either acutely or chronically infectious and diseased divided by the number of all pigs per all days in the model) to variation in the transition parameter β, when simultaneously changed for all age groups by multiplication with a factor between 0.2 and 2

**Fig. 5:**
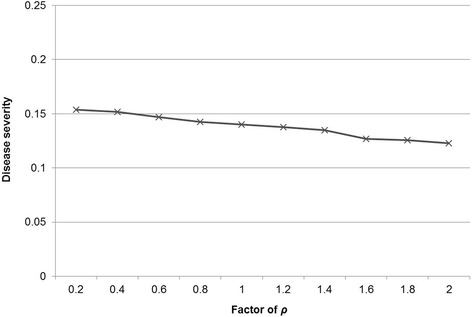
Evaluation of the sensitivity of the outcome variable (disease severity = number of pigs per day either acutely or chronically infectious and diseased divided by the number of all pigs per all days in the model) to variation in the transition parameter ρ, when simultaneously changed for all age groups by multiplication with a factor between 0.2 and 2

The sensitivity of the model to variation in the transition parameters *β* and *ρ* was assessed. The overall outcome “disease severity” was strongly influenced by the level of *β*, when simultaneously changed for all age groups by multiplying with values between 0.2 and 2 (Fig. 4), but not influenced by *ρ*, when simultaneously changed for all groups with factors ranging from 0.2 to 2 (Fig. 5).

### Impact of different factors

Overall, 18 different scenarios reflecting different combinations of risk and protective factors were tested (Table 2). Actually, the number of possible scenarios would have been 32 (*n* = 2^f^ with f = number of factors; *n* = 2^5^ = 32), but taking into account that in more than 70 % of pig herds in Europe vaccination against *M. hyopneumoniae* is routinely applied to suckling pigs [2], it was decided to use only two scenarios without vaccination. As a result, the number of scenarios dropped from 32 to 18 (*n* = 2^4^ [all scenarios with vaccination] + 2 [specific scenarios without vaccination]).

**Fig. 6:**
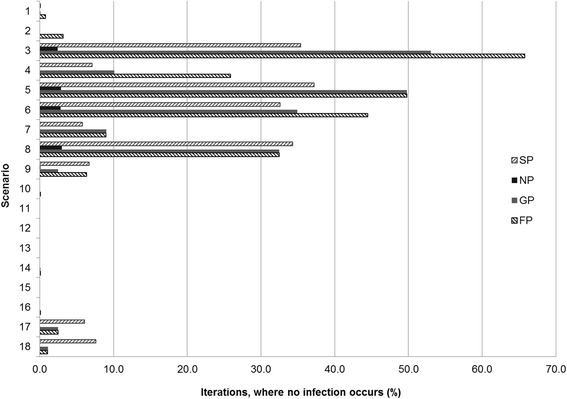
Summary of simulations that did not lead to transmission of infection in 18 scenarios of a compartmental mathematical model of within-herd transmission of *M. hyopneumoniae* (SP = suckling pigs; NP = nursery pigs; GP = growing pigs; FP = finishing pigs)

The lowest ‘disease severity’ was observed under scenario #3, where gilts are in contact with live animals during their acclimatisation, piglets suckle for 21 days and are vaccinated against *M. hyopneumoniae*, fattening pigs do not have contact with other age groups during (re-)stocking of compartments and are not suffering from co-infections. Under this scenario, pigs become either acutely or chronically diseased and infectious for 0.3 % of their lifetime as determined by estimated ‘disease days’ and ‘pig days’, i.e. the ‘disease severity’. In 35.4 % of all simulations of scenario #3 no spread of infection was observed among suckling pigs (Fig. 6). Corresponding figures for nursery, growing and finishing pigs were 2.4 %, 53.0 % and 65.8 %, respectively.

The highest ‘disease severity’ over the whole period was observed in scenario #18. In this scenario gilts do have contact with live animals during their acclimatisation, but suckling pigs are weaned first after 28 days and do not receive vaccination against *M. hyopneumoniae*, fattening pigs have contact with pigs of other ages during (re-)stocking of compartments and pigs are suffering from co-infections. Pigs in this scenario become diseased and either acute or chronic infectious for 26.1 % of their lifetime (Table 2). In 7.6 % of all simulations, there is no transmission of the infection in suckling pigs. Among growing and finishing pigs, this was the case in 1.1 % of all simulations (Fig. 6).

When analysing the detailed results of the different scenarios (Table 2), it was observed that scenario #3 revealed the lowest disease severity in all age groups. In contrast, the highest values for ‘disease severity’ in each particular age group were distributed among two scenarios per age group (#12, #13, #17 and #18).

Multinomial regression analysis was performed in order to compare ‘disease severity’ over the whole lifetime of pigs for the different scenarios (Table 2) with the value of scenario #3 as a baseline. Significant differences were observed for all scenarios, when compared to #3 (*p* < 0.001).

#### Suckling pigs

In scenario #12, suckling pigs were either acutely or chronically diseased and infectious during 2.6 % of their time, this being slightly more than suckling pigs in scenarios #10, #11 and #13. These four scenarios were the only ones, where gilts did not have contact with live animals during their acclimatisation and the suckling period was 28 days compared with 21 days in other scenarios.

#### Nursery and growing pigs

In scenarios #12 and #13 comparably high values for ‘disease severity’ in nursery and growing pigs were found with highest figures being 23.0 % and 8.4 %, respectively. In both scenarios gilts had no contact with live animals during their acclimatisation and the suckling period was 28 days compared with 21 days, and co-infections were present.

#### Finishing pigs

The oldest age group was most affected in terms of ‘disease severity’, when gilts had contact with live animals during their acclimatisation and the suckling period was 28 days compared with 21 days and pigs were not vaccinated against *M. hyopneumoniae* and co-infections were present, and growing and/or finishing pigs had contact with pigs of other age groups during (re-) stocking of compartments (scenario #18). These finishing pigs demonstrated an average of 34.4 % of ‘diseased days’.

**Fig. 7:**
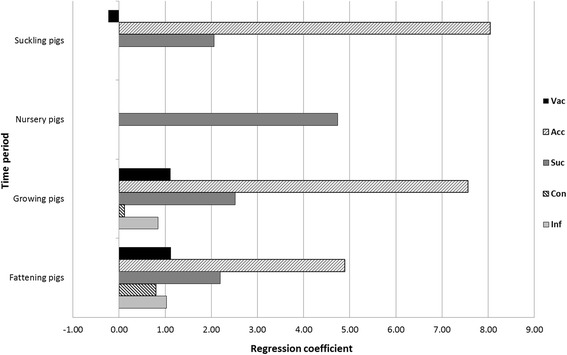
Regression coefficients describing the impact of different risk and protective factors on the ‘disease severity’ in a compartmental mathematical model of within-herd transmission of *M. hyopneumoniae* (SP = suckling pigs; NP = nursery pigs; GP = growing pigs; FP = finishing pigs; Con & Inf for SP and NP have been dropped from the model due to *P* > 0.05)

The results of the logistic regression showed that vaccination of suckling pigs was influential for ‘disease severity’ in growing and fattening pigs, but not in suckling and nursery pigs (Fig. 7). Lack of contact between gilts and other live pigs during the acclimatisation significantly influenced the ‘disease severity’ in suckling pigs and less in growing and finishing pigs. The length of the suckling period equally affected the severity of the disease in all age groups with nursery pigs demonstrating the strongest association. The contact between finishing pigs and pigs of other age groups (i.e. growing pigs or finishing pigs in another compartment) influenced the course of infection among finishing pigs, but not among pigs of other age groups. Finally, the presence of co-infections was associated with higher values for ‘disease severity’ in growing and fattening pigs, but not in other age groups.

When analysing the impact of single risk or protective factors, the whole setting of the particular scenario needs to be considered. For example, the impact of co-infections on the overall disease severity for the whole lifetime was more significant when vaccines were applied to piglets, acclimatisation was performed, suckling period was only 21 days, and contact between fattening pigs of different age was possible (Vac[P], Acc[P], Suc[P], Con[N]). With this setting, the disease severity was 0.02 in case of co-infections being absent (scenario #5) and 0.20 in case of co-infections being present (scenario #8), which reflects a 10-times increase. In a slightly different setting where acclimatisation is not performed properly but everything else is identical (scenario #14 vs. #15) the disease severity elevates from 0.12 without co-infections to 0.24 with co-infections, which is ‘only’ a 2-times increase.

Noteworthy, a reduction of disease severity from particular scenarios is reached by introducing a risk factor instead of eliminating. For example, in scenario #17 vaccination is applied to piglets and gilts have contact to live animals during acclimatisation, though ending up with a disease severity score of 0.25 for the whole lifetime. When skipping the acclimatisation (scenario #13) the disease severity score will be reduced by 20 % to 0.20 for the whole lifetime. This effect is due to an ‘earlier’ spread of the infection, which leads to more recovered and, thereby, no longer susceptible animals in the fattening units.

## Discussion

A stochastic compartmental model of within-herd transmission of *M. hyopneumoniae* was developed and used to assess the impact of different risk and protective factors and to compare potential control measures. The results showed that appropriate gilt acclimatisation, short suckling period and maintenance of all-in-all-out procedure in all steps of production are the most important management factors for reducing within-herd transmission of *M. hyopneumoniae*. Farmers and veterinarians can carefully use this model and specifically the table with the outcomes of different scenarios to explore the most crucial points in a particular pig herd, where frequent transmission of *M. hyopneumoniae* occurs.

The flexible model described in this study can be adapted to assess the transmission of the pathogen in different herd management systems, e.g. variation in the duration of suckling period, contact between pigs of different age etc. Therefore, the model – considering the specific conditions - can be used in different industry settings, and can help to investigate the impact of control measures in different production systems. In order to facilitate such wider use of the model, the open source software ‘R’ was used as software platform for coding and simulations. The entire R code is available from the authors on request.

Some assumptions, like random mixing of all animals in each batch, might deviate results of the model from the reality, but they had to be made with regard of simplicity. The R code in its current form requires already a significant amount of computing power and would no longer run on a standard personal computer in less than 24 h, when including more steps of modelling, e.g. contact structure considering different litters of suckling pigs, etc.

Moreover to this simplification, the model has been parameterised by considering default production values from Germany (e.g. baseline mortality in particular age categories, etc.) and thus is limited to similar industry settings. It cannot be ruled out that in extremely different settings, e.g. backyard farming in Asia, the outcomes of this model do not apply and request a careful interpretation. This also applies to transition parameters calculated from other studies. There is always a specific (farm) setting, i.e. management, behind the outcome, that can have an impact on the data and thereby could have influence our assumptions, e.g. the *β* for each age group, which was calculated from observed increases of prevalence in a longitudinal study [13]. Similar, the experts might have over- or underestimated the multiplying factor for *β* when asked for the impact of co-infections on this figure. Finally, the model does neither account for multiple infections with several strain of *M. hyopneumoniae* nor for virulence of the one or more strains in particularly infected herds. Infections with more than one strain have been reported [34] and also virulence might vary significantly [1], but lack of detailed information about impact on parameters and a tremendous increase of computing power needed to account for these factors in the model precluded further consideration. It is assumed that in herds infected with a low virulent strain, all effects will be smaller, whereas in a herd infected with a high virulent strain all effects might be more sever. The same applies to the potential number of strains.

The model described here is complementary to an earlier study [35]. The latter focused on the veterinarian’s view on the severity of EP and how this view changes with the increased availability of consistent scientific evidence, whereas our model focuses on the course of infection and the impact of intervention strategies. There is no direct link between the two models and it remains questionable, whether there is an option to establish such an interface, because the aim and scope of both models are quite diverse.

### Validation and model sensitivity

The model was tested for internal and external validity. No ‘unexpected’ model behaviour was observed, and all outcomes, especially when testing and analysing ‘extreme scenarios’, were consistent with published data and were biologically sound. Similar attempts of model validation, i.e. comparison of data and evaluation of biological soundness, have been described previously for other models estimating the transmission of PRRSv [17] and *Salmonella typhimurium* [18] in pig herds.

Instead of a deterministic model, a stochastic approach was chosen in order to account for biologically occurring variation in pig populations and the course of *M. hyopneumoniae* infection. In the early stages of the epidemic, single exposed pigs might die by chance (e.g. crashed by the mother dam) prior to transmitting the pathogen to a pen-mate, thereby leading to extinction of transmission. This variation of the course of infection is consistent with reports about different infection and disease pathogenesis patterns [33]. The stochastic approach chosen here also accounts for variation that can occur between herds.

The model was analysed for its sensitivity to assumptions in transition parameters. This was conducted to better assess model robustness and to better understand the course of *M. hyopneumoniae* infection in the model population. This sensitivity analysis focused on the most important parameters of the model only, as has been done in other published studies [18, 36]. Specifically, the transition parameter β was selected for sensitivity analysis, because it is hypothesised that most of the ‘risk factors’ influence this parameter and, thus, knowledge of its sensitivity towards changes is of greatest importance. Moreover, the transition parameter ρ was subjected to a specific sensitivity analysis, because its values were based on expert opinion rather than published literature. No other transition parameters were considered in the sensitivity analysis, as it was felt that a focussed analysis would then have been very difficult given the likely increased variation in output values.

### Impact of different risk and protective factors

Overall, 18 scenarios based on the combination of five different risk and protective factors were analysed in this study. Based on the outcome of the iterations within each of the 18 scenarios, it was confirmed that gilts are the most influential factor for *M. hyopneumoniae* infection levels in suckling pigs. This is reflected in the percentage of iterations without any spread of infection, which was high in all scenarios, where gilts were assumed to have been subjected to appropriate acclimatisation with contact to other live pigs (scenarios 3–9 and 17–18). This observation regarding gilt acclimatisation was further confirmed by the results of the regression analysis, where the acclimatisation of gilts was the most important risk factor for a high ‘disease severity score’ among suckling, growing and finishing pigs. Similar importance of gilts for the course of the infection has been described in other studies determining the prevalence of *M. hyopneumoniae* in suckling pigs and corresponding risk factors, respectively [27, 37]. The hypothesis of young breeding animals being the main source of shedding of *M. hyopneumoniae* in sow herds is underlined by the observation that prevalence of the infection is significantly higher in those herds, where no acclimatisation for replacement boars (i.e. teaser boars) is undertaken [8].

The length of the suckling period was the most important factor with regard to *M. hyopneumoniae* infection in nursery pigs in our model. In longitudinal studies it was shown that the prevalence of *M. hyopneumoniae* infections increases during the suckling period in both sows [22] and suckling pigs [38]. Taking this into account the reason for the success of ‘segregated early weaning’ in order to create pig populations free of *M. hyopneumoniae* [39] becomes apparent. Especially during the first days *post-partum* the likelihood of transmitting the pathogen from sows to their offspring seems to be very low, whereas there is an exponential increase of the chance towards the end of the three to five week period of suckling. Subsequently there is no further transmission between piglets, since infected piglets are now in the incubation period. Thus, the number of nursery pigs infected is mainly driven by infection through their dams.

Considering the overall impact on the disease, the all-in-all-out principle is the most important measure for preventing the transmission of *M. hyopneumoniae* in closed pig herds. The importance of separating age groups has been described numerous times [2, 3, 40]. The feedback, i.e. increasing infection pressure, was modelled as a random contact between the oldest finishing pigs and a group of growing pigs at any point in time during their production stage. On the particular day of contact, the likelihood for the growing pigs to become exposed (i.e. force of infection) was not only influenced by the number of growing pigs in I_a_ and I_c_, but also by the number of fattening pigs in I_a_ and I_c_. The effect of these scenario characteristics is long term, significantly higher than the short-term effect among the first batch of growing pigs. This could explain, why after reaching a steady state of ‘high transmission rates followed by acute and chronic infectiousness followed by high transmission in the next contact batch there is such a huge impact of the break achieved by the all-in-all-out risk management approach. These findings appear to be biologically sound and consistent with observations made for other pathogens, giving confidence in the model structure and its outputs [17, 18].

The observation that ‘vaccination’ had only marginal impact on the disease may be attributed to the limits of current vaccines against *M. hyopneumoniae*. They neither prevent from infection, nor do protect the animals 100 % from disease, when applied very early in pigs’ life [15]. Moreover, only the aspect of reduction in the transmission rate was considered in the model, whereas a potential shortening of the time of being infectious could not be taken into account due to a lack of detailed information. The same facts apply to maternally derived immunity comprised by cells and specific antibodies that piglets receive from their dam. It is possible that both influence the infectious process by reducing clinical expression of EP in such piglets and thereby reducing the infectious period for the same animals. However, the effect is supposed being marginal, no robust data is available and therefore, this has not been considered in the model.

## Conclusion

The output produced by this stochastic compartmental mathematical model of within-herd transmission of *M. hyopneumoniae* allows comparison of different prevention programmes and strategies for controlling EP. The identified intervention measures, namely appropriate acclimatisation of gilts, short suckling period and implementation of the all-in-all-out approach, will result in reduction of prevalence and, thus in improved pig health and welfare as well as a considerable reduction in antimicrobial usage. If combined with economic calculations, the model can provide a practical tool for informing decisions for specific herds.

## Additional files

Additional file 1: **Line diagram of animals per compartment.** Line diagram describing the most likely course of a *M. hyopneumoniae* infection in a batch of 293 pigs, when three protective factors are all present (Vac[P], Acc[P], Suc[P]) and two risk factors are both absent (Con[N], Inf[N]). Lines represent the average of 1,000 iterations of the stochastic compartment model. (TIF 178 kb)

Additional file 2: **Box plot of disease secerity.** Box plots describing the severity of a M. hyopneumoniae infection in a batch of 293 pigs, when three protective factors are all present (Vac[P], Acc[P], Suc[P]) and two risk factors are both absent (Con[N], Inf[N]). Values represent results of 1,000 iterations of the stochastic compartment model. (TIF 67 kb)

Additional file 3: **Diagram showing convergence of the outcome variable.** Evaluation of the convergence of the outcome variable (disease severity) for an example scenario after several iterations with randomly selected input parameters for the binomially distributed elements (i.e. probability of transition between compartments). (TIF 300 kb)
