# Supplementary figures and images for: Modelling the within-herd transmission of Mycoplasma hyopneumoniae in closed pig herds
Source: Porcine Health Manag. 2016 Apr 1;2:10. doi: 10.1186/s40813-016-0026-1 (PMC5382396; doi:10.1186/s40813-016-0026-1)

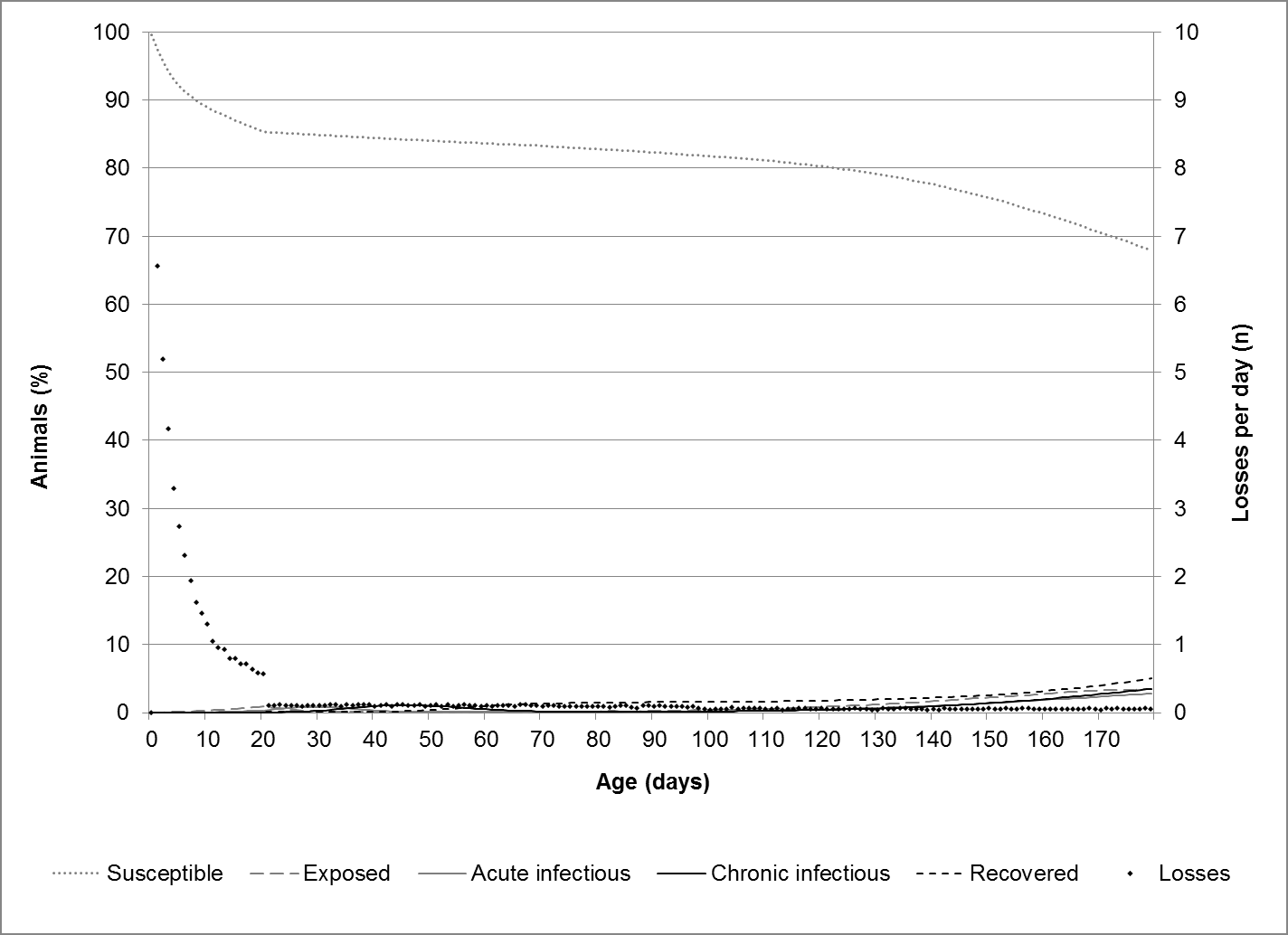

Supplement: Supplementary file 1 — Line diagram of animals per compartment. Line diagram describing the most likely course of a M. hyopneumoniae infection in a batch of 293 pigs, when three protective factors are all present (Vac[P], Acc[P], Suc[P]) and two risk factors are both absent (Con[N], Inf[N]). Lines represent the average of 1,000 iterations of the stochastic compartment model. (TIF 178 kb) [file 40813_2016_26_MOESM1_ESM.tif]

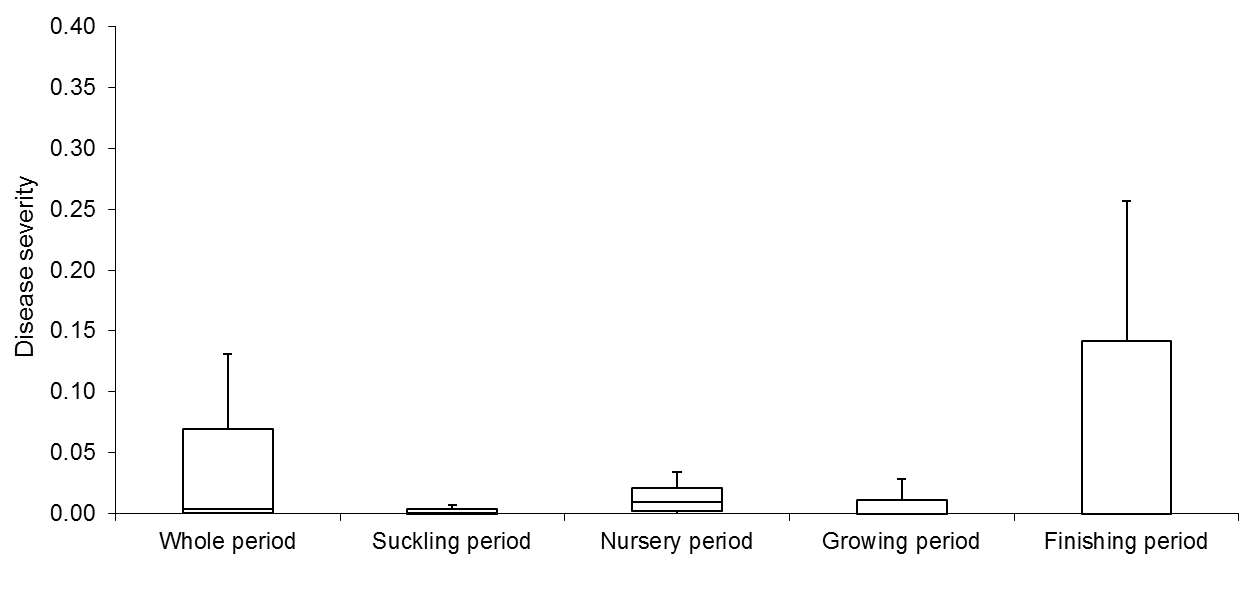

Supplement: Supplementary file 2 — Box plot of disease secerity. Box plots describing the severity of a M. hyopneumoniae infection in a batch of 293 pigs, when three protective factors are all present (Vac[P], Acc[P], Suc[P]) and two risk factors are both absent (Con[N], Inf[N]). Values represent results of 1,000 iterations of the stochastic compartment model. (TIF 67 kb) [file 40813_2016_26_MOESM2_ESM.tif]

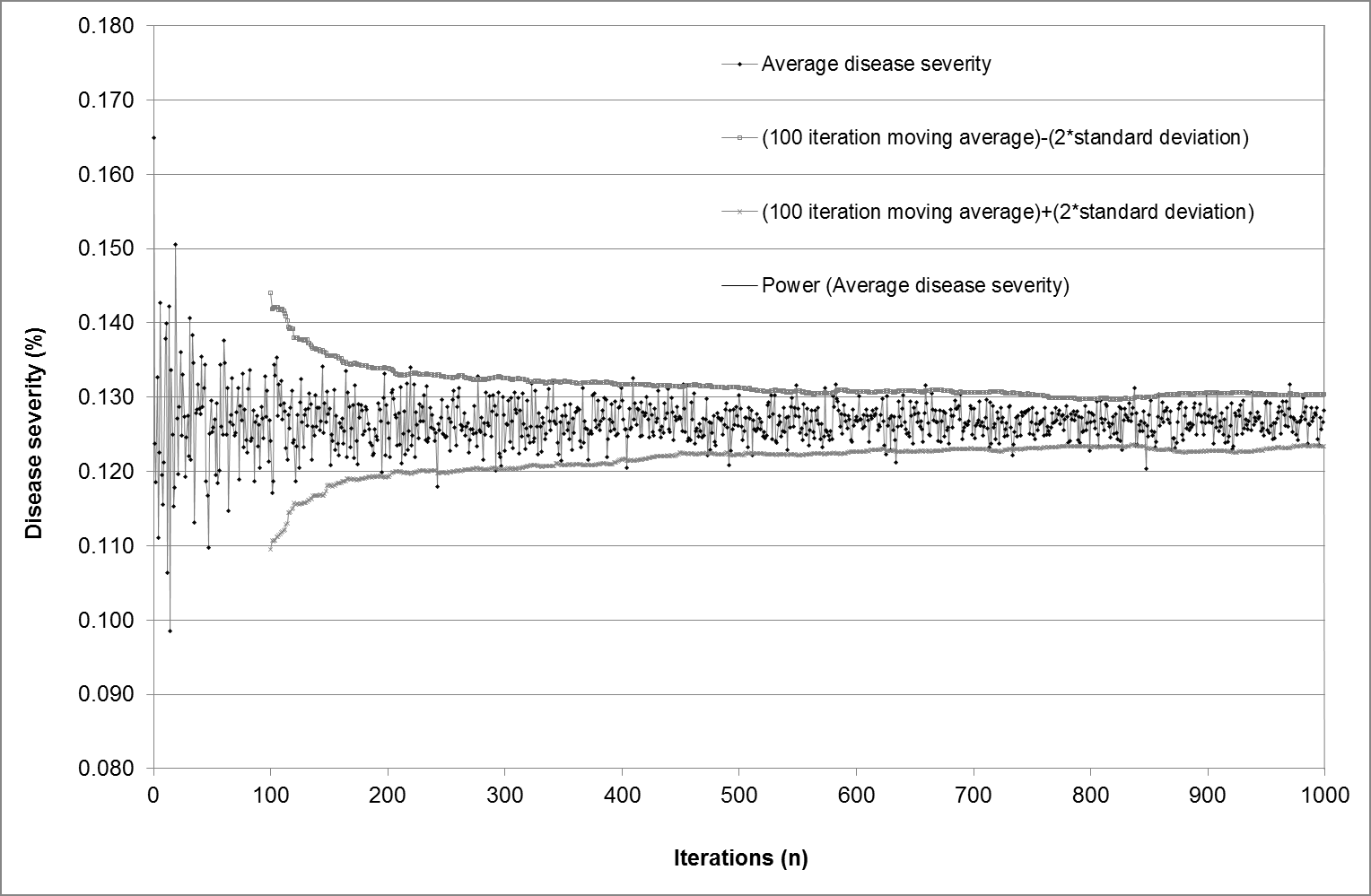

Supplement: Supplementary file 3 — Diagram showing convergence of the outcome variable. Evaluation of the convergence of the outcome variable (disease severity) for an example scenario after several iterations with randomly selected input parameters for the binomially distributed elements (i.e. probability of transition between compartments). (TIF 300 kb) [file 40813_2016_26_MOESM3_ESM.tif]
